# Satisfaction with local healthcare services and medical need among hypertensive patients: a nationwide study

**DOI:** 10.1186/s12889-024-18130-8

**Published:** 2024-03-13

**Authors:** Woorim Kim, Yeong Jun Ju, Soon Young Lee

**Affiliations:** 1 National Hospice Center, National Cancer Control Institute, National Cancer Center, Goyang-si, Gyeonggi-do Republic of Korea; 2 Division of Cancer Control & Policy, National Cancer Control Institute, National Cancer Center, Goyang-si, Gyeonggi-do Republic of Korea; 3 Department of Preventive Medicine and Public Health, Ajou University School of Medicine, 206 World cup-ro, Yeongtong-gu, 16499 Suwon-si Gyeonggi-do, Gyeonggi-do Republic of Korea

**Keywords:** Unmet medical need, Unmet healthcare need, Patient satisfaction, Healthcare accessibility, Hypertension

## Abstract

**Background:**

Investigating the factors associated with unmet medical needs is important since it can reflect access to healthcare. This study examined the relationship between the unmet medical needs of patients with hypertension and their satisfaction with the healthcare services available in their neighborhoods.

**Methods:**

Data were from the 2021 Korean Community Health Survey. The sample included individuals aged 19 years who were diagnosed with hypertension. The main outcome measure was unmet medical need. The relationship between the outcome measure and independent variables were analyzed using multivariate logistic regressions, along with a subgroup analysis based on whether patients were currently receiving treatment for hypertension.

**Results:**

Unmet medical needs were found in 4.3% of the study participants. A higher likelihood of unmet medical needs was found in individuals not satisfied with the healthcare services at proximity (adjusted OR = 1.69, 95% CI: 1.49–1.92) compared to those satisfied with services nearby. Similar tendencies were found regardless of whether individuals were currently receiving treatment for hypertension, although larger differences were found between groups in participants who were currently not receiving treatment.

**Conclusions:**

The findings infer the need to consider patient satisfaction with nearby healthcare services in implementing public health policies that address unmet medical need in patients with hypertension.

## Background

Hypertension is a globally prevalent disease affecting approximately 31% of the adult population [1] and is defined as having a systolic blood pressure of 140 mmHg or higher and/or a diastolic blood pressure of 90 mmHg or higher. Further, hypertension is a prevalent and modifiable risk factor for cardiovascular disease (CVD) and overall mortality [1–3]. A sizable body of evidence suggests a causal relationship between high blood pressure and CVD, highlighting the importance of managing hypertension. CVD is reported as one of the most serious health problems and the leading cause of mortality worldwide [4, 5]. Overall, high blood pressure and its related complications are estimated to account for approximately 10% of global healthcare spending, increasing the financial burden on healthcare systems [6, 7].

The care cascade for hypertension (i.e., awareness, treatment, and control) has been proposed as a framework for improving the management of blood pressure [8]. Despite improvements in hypertension awareness, treatment, and control rates, along with techniques for controlling blood pressure, notably pharmacotherapy and lifestyle interventions, hypertension management remains a challenge for healthcare systems in many countries [9, 10]. Unmet medical needs are the difference in the medical services required to manage a health-related problem (e.g., hypertension) and the services received by patients, and the factors associated with these unmet medical needs for patients with hypertension require investigation [11]. Since unmet medical needs are an important measure of healthcare systems that reflect how patients subjectively assess their access to healthcare, they may be affected by factors such as the cost of visiting clinics or hospitals [12, 13]. Further, experiencing unmet medical needs has been related to poor health outcomes and may contribute to increasing health disparities [14].

Individuals’ satisfaction with the medical services available in their neighborhoods may correlate with the likelihood of experiencing unmet medical needs in the treatment of patients with chronic diseases, including hypertension, as successful management requires regular care and high treatment adherence [15]. As having access to high-quality medical care is related to patient satisfaction, it is also associated with the unmet medical needs of patients with hypertension [16]. Therefore, this study aimed to investigate the association between the unmet needs of patients with hypertension and their satisfaction with the healthcare services available in their neighborhoods. Further, we conducted a subgroup analysis according to the current receipt of treatment for hypertension.

## Methods

### Study design

This study used the 2021 Korea Community Health Survey (KCHS) data. As the KCHS data are cross-sectional in nature, a cross-sectional design was applied to investigate the association between the unmet needs of patients with hypertension and their satisfaction with nearby healthcare services. The final study population included 50,068 adults aged 19 years or above that have been diagnosed of hypertension by a physician, which was measured based on self-reports of the study participants.

### Data source and collection

The 2021 KCHS data were used for this study. The KCHS are cross-sectional data collected through annual surveys performed by the Korea Disease Control and Prevention Agency (KDCA) using the Computer Assisted Personal Interviewing (CAPI) method. The study sample is selected using stratified, multistage, probability-cluster sampling on the national survey data and hence, the sample can be seen as a representative of the Korean population [17]. The KCHS data contains various information on the sociodemographic, economic, and health related factors of the participants.

### Data management and variables

The outcome and independent variables were extracted from the retrieved KCHS data for analysis. The outcome variable was the experience of unmet medical needs. Self-reported unmet medical needs were measured using the question, “In the past 1 year, were you in need of receiving a diagnosis or treatment in a clinic or hospital (excluding a dental clinic) but were unable to receive one? Responses to the questions were “yes,” “no,” and “not applicable (did not need a diagnosis or treatment.” Those who responded “yes” were identified as having unmet medical need.

The independent variable of interest was satisfaction level with the healthcare services available in the study participants’ neighborhoods. This variable was measured based on self-reports to the phrase “I am satisfied with the medical services (public health centers, clinics, hospitals, traditional Korean clinic and hospitals, and pharmacies)” available in our neighborhood.” Available responses were a “yes” or a “no” in which individuals were categorized as “satisfied” or “not satisfied” accordingly.

The covariates included in this study were sex, age group, educational level, income, occupation, obesity, subjective health status, perceived stress status, depressive symptoms, current smoking, monthly drinking, moderate-to-vigorous physical activity, diabetes, current hypertension treatment status, and region of residence. The Body Mass Index (BMI) was used to categorize body weight (i.e., <18.5, underweight status; 18.5–24.9, normal weight; ≥25.0, overweight or obese status). The Korean version of the Patient Health Questionnaire-9 (PHQ-9) assessed participants’ depressive symptoms. As suggested in previous studies, a cutoff score of 10 was used to indicate depressive symptoms. In the KCHS, individuals were asked by a physician whether they had been diagnosed with hypertension by a physician. Individuals who replied that they were diagnosed with hypertension were asked whether they were currently receiving treatment. Current hypertension treatment status was measured based on whether the study participants were currently receiving pharmacological treatment for at least 20 days.

### Data analysis

The general characteristics of the study participants were examined using chi-square tests. Multivariate logistic regression was conducted to study the association between unmet medical needs and satisfaction with the healthcare services available in the participants’ residential neighborhoods. Additionally, a subgroup analysis was performed based on the receipt of treatment for hypertension. All analyses were conducted with adjustments for all covariates, namely sex, age group, educational level, income, occupation, obesity, subjective health status, perceived stress status, depressive symptoms, current smoking, monthly drinking, moderate-to-vigorous physical activity, diabetes, current hypertension treatment status, and region of residence. The results are presented as adjusted odds ratios (OR) and their 95% confidence intervals (95% CI). P-values were two-sided and significant at *P* < 0.05. Analyses were performed using the SAS software (version 9.4 SAS Institute, Cary, NC, USA).

## Results

The main results of the analysis on the association between medical needs and satisfaction with healthcare services available in the study participants’ residential neighborhoods are presented in Tables 1 and 2. The results of Table 1 shows that unmet medical needs were more prevalent in individuals not satisfied with nearby healthcare services than those who are satisfied. Similar tendencies can be found in Table 2, which reveals confirms the investigated association as individuals not satisfied with nearby healthcare services show a higher likelihood of reporting unmet medical need.

**Table 1:** General characteristics of the study subjects

Variables	Total		Unmet medical need				P-Value
			Yes		No		
	N	%	N	%	N	%	
**Satisfaction with healthcare services available at residing neighborhood**							
Yes	39,070	82.4	1,572	3.7	37,498	96.3	< 0.001
No	10,998	17.6	837	7.1	10,161	92.9	
**Sex**							
Male	22,401	50.7	813	3.5	21,588	96.5	< 0.001
Female	27,667	49.3	1,596	5.2	26,071	94.8	
**Age group**							
19–29	228	1.0	20	8.4	208	91.6	< 0.001
30–39	727	2.5	44	5.6	683	94.4	
40–49	2,807	8.8	135	4.6	2,672	95.4	
50–59	7,662	20.5	368	4.7	7,294	95.3	
60–69	14,665	28.7	662	4.3	14,003	95.7	
70+	23,979	38.5	1,180	3.8	22,799	96.2	
**Education level**							
None	8,458	10.2	631	7.0	7,827	93.0	< 0.001
Elementary school	12,691	19.7	648	4.7	12,043	95.3	
Middle school	8,701	17.1	362	3.9	8,339	96.1	
High school	12,625	30.2	515	4.2	12,110	95.8	
College or above	7,593	22.8	253	3.3	7,340	96.7	
**Income**							
Low	13,462	18.2	957	7.0	12,505	93.0	< 0.001
Low-middle	11,786	19.9	542	4.1	11,244	95.9	
Middle	7,692	16.1	326	4.1	7,366	95.9	
Middle-high	5,767	13.5	206	3.4	5,561	96.6	
High	11,361	32.3	378	3.4	10,983	96.6	
**Occupation**							
Professional or administrative position	2,448	7.5	76	3.0	2,372	97.0	< 0.001
Office work	1,705	5.4	51	2.7	1,654	97.3	
Sales and service	4,770	10.5	227	5.1	4,543	94.9	
Agriculture and fishery	4,940	3.4	273	5.3	4,667	94.7	
Blue collar work or simple labor	10,752	21.9	518	4.0	10,234	96.0	
Unemployed	25,453	51.3	1,264	4.6	24,189	95.4	
**Body weight**							
Underweight	1,450	2.1	129	6.7	1,321	93.3	0.0009
Normal	29,188	55.6	1,352	4.0	27,836	96.0	
Overweight or obese	19,430	42.2	928	4.5	18,502	95.5	
**Subjective health status**							
Fair	12,411	27.3	316	2.4	12,095	97.6	< 0.001
Poor	37,657	72.7	2,093	5.0	35,564	95.0	
**Perceived stress**							
Yes	9,660	21.7	910	8.3	8,750	91.7	< 0.001
No	40,408	78.3	1,499	3.2	38,909	96.8	
**Depressive symptoms**							
No	47,809	95.2	2,031	3.7	45,778	96.3	< 0.001
Yes	2,259	4.8	378	16.6	1,881	83.4	
**Current smoking**							
Yes	6,177	14.5	306	4.9	5,871	95.1	0.0532
No	43,891	85.5	2,103	4.2	41,788	95.8	
**Current monthly drinking**							
Yes	16,215	38.3	681	4.1	15,534	95.9	0.2898
No	33,853	61.7	1,728	4.4	32,125	95.6	
**Moderate-to-vigorous physical activity**							
No	42,765	85.6	2,035	4.4	40,730	95.6	0.4262
Yes	7,303	14.4	374	4.1	6,929	95.9	
**Diabetes**							
No	36,391	73.4	1,811	4.5	34,580	95.5	0.0571
Yes	13,677	26.6	598	3.9	13,079	96.1	
**Receiving medical treatment for hypertension**							
No	2,099	6.2	159	7.1	1,940	92.9	< 0.001
Yes	47,969	93.8	2,250	4.1	45,719	95.9	
**Residing region**							
Urban	25,484	78.5	1,028	4.1	24,456	95.9	0.0003
Rural	24,584	21.5	1,381	5.0	23,203	95.0	
Total	50,068	100.0	1,765	4.3	53,438	95.7	

**Table 2:** The association between unmet medical need and satisfaction status with healthcare services available at residing neighborhood

Variables	Unmet medical need			
	Adjusted Odds Ratio (OR)	95% Confidence Interval		
**Satisfaction with healthcare services available at residing neighborhood**				
Yes	1.00			
No	1.69	1.49	–	1.92
**Sex**				
Male	1.00			
Female	1.32	1.14	–	1.54
**Age group**				
19–29	1.00			
30–39	0.80	0.43	–	1.49
40–49	0.77	0.43	–	1.37
50–59	0.83	0.48	–	1.45
60–69	0.65	0.37	–	1.14
70+	0.41	0.23	–	0.72
**Education level**				
None	1.00			
Elementary school	0.70	0.59	–	0.84
Middle school	0.56	0.45	–	0.68
High school	0.61	0.50	–	0.75
College or above	0.53	0.41	–	0.68
**Income**				
Low	1.00			
Low-middle	0.68	0.57	–	0.80
Middle	0.65	0.53	–	0.79
Middle-high	0.50	0.40	–	0.63
High	0.51	0.42	–	0.62
**Occupation**				
Professional or administrative position	1.00			
Office work	0.87	0.54	–	1.41
Sales and service	1.45	1.03	–	2.05
Agriculture and fishery	1.71	1.20	–	2.44
Blue collar work or simple labor	1.29	0.94	–	1.77
Unemployed	1.29	0.94	–	1.77
**Body weight**				
Underweight	1.00			
Normal	0.80	0.61	–	1.05
Overweight or obese	0.88	0.66	–	1.16
**Subjective health status**				
Fair	1.00			
Poor	1.57	1.33	–	1.84
**Perceived stress**				
Yes	1.00			
No	0.53	0.46	–	0.60
**Depressive symptoms**				
No	1.00			
Yes	2.92	2.47	–	3.46
**Current smoking**				
Yes	1.00			
No	0.90	0.75	–	1.09
**Current monthly drinking**				
Yes	1.00			
No	0.90	0.78	–	1.03
**Moderate-to-vigorous physical activity**				
No	1.00			
Yes	1.06	0.90	–	1.25
**Diabetes**				
No	1.00			
Yes	0.80	0.71	–	0.91
**Receiving medical treatment for hypertension**				
No	1.00			
Yes	0.61	0.48	–	0.77
**Residing region**				
Urban	1.00			
Rural	0.97	0.86	–	1.09

Specifically, the general characteristics of the participants are shown in Table 1. Unmet medical needs were found in 1,765 (4.3%) of 50,068 individuals diagnosed with hypertension. A total of 39,070 (82.4%) participants were satisfied with the healthcare services available in their neighborhood, whereas 10,998 (17.6%) participants were not satisfied. Unmet medical needs were more prevalent in the “non satisfied” group (7.1%) compared to the “satisfied” group (3.7%).

The results of the multivariable logistic regression analysis are revealed in Table 2. Individuals who were not satisfied with the healthcare services in their neighborhoods were more likely to have unmet medical needs (adjusted OR = 1.69, 95% CI: 1.49–1.92) compared to individuals satisfied with healthcare services available nearby. Lower odds of unmet medical needs were found in individuals currently receiving treatment for hypertension compared to those not receiving treatment.

The results of the subgroup analysis based on the current hypertension treatment status are shown in Table 3. The patterns observed in the main findings were generally maintained, regardless of the current hypertension treatment status. However, larger differences were found between the two groups according to the satisfaction reported by individuals who were not receiving treatment (adjusted OR = 2.50, 95% CI: 1.89–3.30) compared to those currently receiving treatment (adjusted OR = 1.64, 95% CI: 1.44–1.87).

**Table 3:** Results of the subgroup analysis conducted based on current hypertension treatment status

Variables		Unmet medical need			
		Adjusted Odds Ratio (OR)	95% Confidence Interval		
**Receiving medical treatment for hypertension**	**Satisfaction with healthcare services available at residing neighborhood**				
**No**	Yes	1.00			
	No	2.50	1.89	–	3.30
**Yes**	Yes	1.00			
	No	1.64	1.44	–	1.87

## Discussion

This study examined the relationship between the unmet medical needs of patients with hypertension and their satisfaction with healthcare services available in their neighborhoods. The current results showed that the likelihood of experiencing unmet medical needs was related to participants’ satisfaction with the healthcare services available in their neighborhoods. Similar patterns of relationships were observed between those patients currently receiving and those not receiving treatment for hypertension, although the difference between individuals who were satisfied and unsatisfied with the available local healthcare resources and in the likelihood of experiencing unmet needs was magnified in those currently not receiving treatment. These findings suggest the importance of patient satisfaction with existing healthcare services in their neighborhood in addressing the unmet medical needs of patients with hypertension.

Several factors have previously been identified as determinants of unmet medical needs, which have also been accounted for in the analysis investigating the association between unmet medical need and satisfaction with healthcare services available nearby in patients with hypertension. A recognizable factor is socioeconomic status, as individuals with lower income or education are more likely to report unmet medical needs [18, 19]. Further, having a low income was associated with increased unmet medical needs, even in countries with universal health coverage [20]. Accordingly, the findings also reveal an association between lower income or education level and a higher likelihood of experiencing unmet medical need. In addition, employment status has been identified as a possible influencing factor, as comparatively vulnerable individuals, including precarious workers, report increased unmet medical needs [21]. In this study, workers in sales and service or agriculture and fishery reported higher levels of unmet healthcare need. Such tendencies may have been influenced by the fact that workers in the cited industries tend to report longer work hours, which in turn has been related to unmet need for accessing healthcare facilities [22–24]. Lastly, females were more likely to report unmet healthcare need, as in that in the results of previous studies [25].

Interestingly, chronic medical conditions have been previously related to unmet medical needs, highlighting that unmet medical needs indicate inadequate access to healthcare when the appropriate management of chronic diseases requires regular, ongoing care [26]. Healthcare access is particularly critical because inadequate access can lead to poor health outcomes and increased health disparities [14]. The current findings provide important insights by indicating that satisfaction with the availability of healthcare services in one’s neighborhood is associated with the likelihood of perceived unmet medical needs in patients with hypertension. Patients who are dissatisfied with the locally available services may experience barriers in regularly accessing care in healthcare institutions and facilities due to the increased distances to travel for treatment [27]. Otherwise, patients lacking the resources to travel further may continuously receive care at nearby institutions but perceive that they have unmet medical needs or show decreased treatment adherence for their hypertension. In fact, previous literature on developing countries cite inadequate services as a barrier to visiting health facilities and report preferring clinics and hospitals that provide better access to specialists and higher quality medications and laboratory testing [28]. Patient satisfaction with available healthcare services has also been associated with increased treatment adherence, and low adherence is a notable concern since it can result in reduced effectiveness and efficiency of pharmacological treatments, which is important for reducing blood pressure and decreasing the risk of CVDs [29–31].

These findings also show that the degree of association between the unmet medical needs of patients with hypertension and satisfaction with locally available healthcare services is magnified among those who currently not receiving hypertension treatment. These results are noteworthy because pharmacological and non-pharmacological treatments are critical for controlling blood pressure and reducing the risk of CVD or mortality in patients with hypertension [32, 33]. Moreover, patient adherence is essential for enhancing blood pressure control and minimizing hypertension-related risks [34]. Considering the importance of hypertension treatment, the potential factors associated with the unmet medical needs of patients who do not receive treatment need to be understood.

This study had some limitations. First, this study was cross-sectional; thus, the analyzed results could not infer causality. Second, although previous studies have shown that unmet medical needs can be adequately assessed through self-report in population-based national surveys, limitations may apply because unmet medical needs were measured solely using self-report [14]. Third, the possibility of residual confounding factors cannot be entirely ruled out, although we adjusted for a number of covariates in the analyses. However, one notable strength of this study is its large random sample of the entire South Korean population, which was extracted from reliable nationwide data. Our current findings are also unique since this study is the first to examine the association between the unmet medical needs of patients with hypertension and their satisfaction with the healthcare services available in their neighborhood.

## Conclusions

In patients with hypertension, unmet medical needs were related to satisfaction with healthcare services in the patients’ neighborhoods. A stronger association was observed among those patients currently receiving treatment for hypertension compared to those who were not being treated. The results indicated the significance of patient satisfaction in the likelihood of reporting unmet medical needs among those patients with hypertension. As the management of hypertension remains a challenge in many countries, in which its importance is increasing owing to population aging and the rise in the number of affected individuals, the findings infer the need to consider patient satisfaction with nearby healthcare services in implementing public health policies that address unmet medical need in patients with chronic disease. An emphasis should be made on ensuring access to appropriate and high-quality care for patients with hypertension.

## Data Availability

Data can be downloaded after application from the Korea Community Health Survey website (https://chs.kdca.go.kr/chs/main.do).
